# Systematic Item Content and Overlap Analysis of Self-Reported Multiple Sleep Disorder Screening Questionnaires in Adults

**DOI:** 10.3390/jcm12030852

**Published:** 2023-01-20

**Authors:** Christophe Gauld, Vincent P. Martin, Alexandre Richaud, Sébastien Baillieul, Lucie Vicente, Jean-Lorrain Perromat, Issa Zreik, Jacques Taillard, Pierre Alexis Geoffroy, Régis Lopez, Jean-Arthur Micoulaud-Franchi

**Affiliations:** 1 Service Psychopathologie du Développement de l’Enfant et de l’Adolescent, Hospices Civils de Lyon & Université de Lyon 1, 69500 Bron, France; 2 Institut des Sciences Cognitives Marc Jeannerod, UMR 5229 CNRS & Université Claude Bernard Lyon 1, 69500 Bron, France; 3 CNRS, Bordeaux INP, LaBRI, UMR 5800, University of Bordeaux, 33400 Talence, France; 4 CNRS, SANPSY, UMR 6033, University of Bordeaux, 33000 Bordeaux, France; 5 University Sleep Clinic, University Hospital of Bordeaux, Place Amélie Raba-Leon, 33076 Bordeaux, France; 6 HP2 Laboratory, INSERM U1042, Grenoble Alpes University, 38400 Grenoble, France; 7 Pôle Thorax et Vaisseaux, Grenoble Alpes University Hospital, 38700 Grenoble, France; 8 Département de Psychiatrie et d’addictologie, AP-HP, GHU Paris Nord, DMU Neurosciences, Hopital Bichat-Claude Bernard, 75018 Paris, France; 9 GHU Paris-Psychiatry & Neurosciences, 1 Rue Cabanis, 75014 Paris, France; 10 NeuroDiderot, Inserm, Université de Paris, FHU I2-D2, 75019 Paris, France; 11 CNRS UPR 3212, Institute for Cellular and Integrative Neurosciences, 67000 Strasbourg, France; 12 Institut des Neurosciences de Montpellier (INM), Université de Montpellier, 34000 Montpellier, France; 13 Unité des Troubles du Sommeil, Département de Neurologie, CHU Montpellier, 34000 Montpellier, France

**Keywords:** sleep disorders, sleep symptoms, questionnaires, content analysis, symptom overlap

## Abstract

Sleep disorders are defined on the basis of diagnostic criteria presented in medical classifications. However, no consensus has emerged on the exact list of operational symptoms that should be systematically investigated in the field of sleep medicine. We propose a systematic analysis of sleep symptoms that figure in a set of self-reported multiple sleep disorder screening questionnaires for adult populations, to identify the content overlap of symptoms that probe the presence of central sleep symptoms, and to highlight the potential level of heterogeneity among sleep disorder questionnaires. The method comprises three steps: (i) the selection of self-reported multiple sleep disorder screening questionnaires; (ii) item extraction and selection; (iii) the extraction of symptoms from items. Frequency of sleep symptoms and content overlap (Jaccard Index) are analyzed. We extracted 469 items that provide 60 different symptoms from 12 questionnaires. Insomnia, somnolence, and sleep-related breathing symptoms were found in all the questionnaires. The mean overlap among all questionnaires evaluated with the Jaccard Index is 0.44, i.e., moderate similarity. Despite limitations related to the selection of questionnaires and the symptom extraction and harmonization, this study underlines the need to standardize sleep symptom contents for sleep medicine in order to enhance the practicability, reliability, and validity of sleep disorder diagnoses.

## 1. Introduction

Sleep disorders are consensually defined on the basis of diagnostic criteria presented in medical classifications. In our analysis of the diagnostic criteria of the International Classification of Sleep Disorders, Third Edition (ICSD-3, proposed by the American Academy of Sleep Medicine [AASM]) [1,2] and of the sleep-wake disorder section of the Diagnostic and Statistical Manual of Mental Disorders, Fifth Edition (DSM-5, proposed by the American Psychiatric Association [APA]) [3,4], the vast majority of the main sleep disorders analyzed presented symptoms in their diagnostic criteria [5]. Symptoms can be defined as clinical manifestations related to a disorder. Among the 43 diagnoses, only five—four sleep-related breathing disorders and one sleep-related movement disorder (periodic limb movement disorder)—do not have any symptom-based criteria [5].

Collecting symptoms is central to diagnosing the main categories and diagnostic subtypes of sleep disorders, differentiating the normal from the pathological, and evaluating their severity [5,6,7,8,9,10]. Moreover, symptom-oriented classifications are considered by clinicians as the most useful since the ICSD-1 [11]. Various attempts have thus been made to develop a standard definition for recognized sleep symptoms in sleep medicine. Both the AASM and the European Sleep Research Society (ESRS) have proposed symptom definitions for insomnia disorder [12,13] and sleep-related breathing disorders [10,14]. Other initiatives have been undertaken to define symptoms for restless leg syndrome [15], central disorders of hypersomnolence [16], parasomnia disorder [17,18], and sleep-related bruxism disorder [19].

Nevertheless, no consensus has emerged on the exact list of operational symptoms that should be investigated systematically in sleep medicine [6]. In the ICSD-3, the terminology used for symptom criteria may lack precision or sufficient specificity and may vary depending on the main categories of sleep disorders [5]. This discrepancy may lead to disharmony between clinicians and thus reduce the practicability (i.e., the efficiency of the clinical manifestation with regard to the clinical reasoning process), reliability (i.e., the inter-rater agreement on identification of a clinical manifestation and agreement over time), and validity (i.e., the relation of the clinical manifestation with the pathophysiological mechanism) of diagnosis in sleep medicine [20].

Empirical research is thus necessary to refine the symptoms that should be investigated in sleep medicine. A consensual terminology of sleep symptoms in sleep medicine should be developed to reduce the variability in how symptoms are considered in the sleep literature and by clinicians [5,8,21]. The first step is to analyze the symptom content of multiple sleep disorder screening questionnaires.

Several self-reported screening questionnaires for multiple sleep disorders are available. A recent systematic review identified seven self-reported multiple sleep disorder screening questionnaires for adult populations that were evaluated for comprehensiveness, brevity, and psychometric quality [22]. Each of these questionnaires assessed at least three different sleep disorders, but their sleep symptom content was not evaluated [22].

An original method for comparing symptom content in questionnaires is to assess the overlap of item content between questionnaires. In 2017, Fried developed a method of content overlap analysis based on the Jaccard similarity coefficient to analyze the overlap between seven widely used self-reported depression questionnaires [23]. He found considerable heterogeneity among the content of the symptoms, thereby paving the way for more reliable and valid questionnaires based on a better-defined set of symptoms. Such symptom content overlap analysis has since been performed for mental pain [24], mood and bipolar disorder [25,26], obsessive-compulsive disorder [27], anxiety disorder [28,29], and neurological soft signs [30]. To our knowledge, however, this analytical method has not been applied to sleep disorder questionnaires. Interestingly, the only systematic analysis of sleep symptoms was performed using the network analysis method developed by Borsboom (2011) [31]. It found that insomnia and somnolence symptoms are central in the reference classification of the ICSD-3, and that other symptoms specific to the main sleep disorder categories are more peripheral in the network analysis [2,3].

This paper uses the method outlined by Fried [23] to perform a systematic analysis of sleep symptoms in a set of self-reported multiple sleep disorder screening questionnaires for adult populations [22]. Moreover, to provide the opportunity for others to produce the same results with custom data, we propose a handy-to-use tool to perform content overlap analysis.

Our focus here is on the content overlap of items that probe the presence of central sleep symptoms (i.e., insomnia and somnolence symptoms), as opposed to items assessing symptoms specific to the main sleep disorder categories. We expect to find overlap for insomnia and somnolence symptoms but some level of heterogeneity among sleep disorder questionnaires. In addition, we hope that during the extraction of symptoms from the questionnaires studied—a step necessary to study symptom overlap between questionnaires—a consensual set of symptoms important to sleep medicine will be identified.

## 2. Materials and Methods

Our method is based on those described in previous content overlap analysis studies [23,24,25,26,27,28,29,30].

### 2.1. Selection of Self-Reported Multiple Sleep Disorder Screening Questionnaires

In their systematic review of self-reported multiple sleep disorder screening questionnaires for adult populations [22], Klingman et al. (2017) found seven questionnaires: the Auckland Sleep Questionnaire (ASQ) [32], the Sleep Symptom Checklist (SSC) [33], the Sleep Disorder Questionnaire (SDQ) [34], the Holland Sleep Disorders Questionnaire (HSDQ) [35], the Iowa Sleep Disturbances Inventory (ISDI) [36], the Global Sleep Assessment Questionnaire (GSAQ) [37], and the Sleep 50 [38]. A self-reported multiple sleep disorder screening questionnaire was defined as “any standardized tool whose authors claim specifically that it measures at least three sleep-wake disorders and is usually fulfilled by the patient himself”. Only self-reported multiple sleep disorder screening questionnaires that had undergone psychometric validation were considered by Klingman et al. [22].

We started with Klingman’s systematic review and searched for all the sources cited in that article and all the recent articles that in turn have cited it. To ensure exhaustivity in the compilation of these questionnaires, we conferred with experts in the area of sleep disorders [28] and searched Google Scholar with the following search terms: [“sleep disorder*” AND (“diagnosis*” OR “scale*” OR “questionnaires*” OR “screen*” OR “psychometric*”)].

The Pittsburgh Sleep Quality Index (PSQI) [39] was not included in Klingman’s systematic review because the authors considered that this self-reported questionnaire should be interpreted by sleep specialists. However, given its widespread use by the sleep community, we decided to include it. Moreover, based on the « Web of Science » citation counts to estimate how frequently tools have been utilized for research and clinical purposes within the past decade, we complemented the list of self-reported multiple sleep disorder screening questionnaires with four tools: the Oviedo Sleep Questionnaire [40], the Basic Nordic Sleep Questionnaire [41], and the two versions of the Sleep Disorders Symptom Checklist developed by Klingman et al. following their systematic review: the Sleep Disorder Symptom Checklist 17 items (SDS-CL-17) [22] and the 25 items (SDS-CL-25) [42]. We thus explored 12 self-reported multiple sleep disorder screening questionnaires.

### 2.2. Extraction and Selection of Items

First, we extracted the total number of items from the 12 self-reported multiple sleep disorder screening questionnaires [32,33,34,35,36,37,38,39,40,41,42]. An item of a questionnaire is considered any element of the questionnaire to which the subject has to respond [20]. Except for the ASQ [32], the PSQI [39], and the OSQ [40], the number of extracted items corresponded to the number of questions. For example, each question corresponds to an item in the SDS-CL-25 [42], so we extracted 25 items from the 25 questions. Regarding the ASQ, the PSQI, and the OSQ, the number of extracted items was different from the number of questions since some questions contain sub-questions. Thus, 19 items were extracted from the 11 questions of the OSQ, 24 items from the 11 questions of the PSQI, and 84 items from the 30 questions and sub-questions of the ASQ. Finally, we extracted a total of 567 items from the 12 questionnaires.

Second, we selected items related to sleep symptoms. Four types of items were identified as not related to sleep symptoms and were classified as not applicable (NA): sociodemographic data, personal and family history, lifestyle, and contextual elements. Finally, we selected a total of 469 items related to symptoms in the 12 questionnaires.

Items extracted and selected are shown in Supplementary Materials S1.

### 2.3. Extraction of Symptoms from Items

The extraction of symptoms from items involved two steps.

#### 2.3.1. First Step: Symptom Extraction from Items within Each Questionnaire

This step was carried out by identifying all symptoms depicted by the items within each questionnaire. The different symptoms extracted from each of the items were split or lumped [2,3]. As a result, all the symptoms from all the items of each questionnaire were extracted.

Two remarks should be made regarding this step:

1. From a methodological point of view, we used a double-blind method based on a panel of medical examiners outside the sleep community to increase reliability. Therefore, in addition to sleep experts (AR, RL, and JAM), this step was also carried out by medical students (LV, JLP, and IZ). This double-blind approach was used to develop large-scale questionnaire studies in psychiatric epidemiology, which led in particular to the constitution of the DSM-III, such as the US Mental Hygiene Movement, the Midtown Manhattan Study, and the Stirling County studies [43,44,45]. In those studies, the opinion of examiners outside the (sleep medicine) community was considered particularly important to avoid as much as possible value-laden choices and the influence of theoretical backgrounds on the fitting of the items of self-reported questionnaires with diagnostic criteria. Examiners’ opinions were then cross-referenced with those of the sleep experts.
2. From a content analysis point of view, we differentiated three kinds of symptoms within the items: compound symptoms, specific symptoms, and idiosyncratic symptoms, according to the terminology used in previous content overlap analysis studies [23,24,25,26,27,28,29,30].
  i. A compound symptom was extracted from an item referring simultaneously to at least two distinct symptoms. For instance, in the SDS-CL-25, item 6, “I am tired, fatigued, or sleepy during the day,” refers to both symptoms “Fatigue” and “Daytime sleepiness.”
  ii. A specific symptom was extracted from an item that refers specifically and solely to it. For instance, item 10 of the SDS-CL-25 “I snore” refers only to the “Snoring” symptom. When the same symptom was extracted from items referring to it in a specific way and in a compound way, we considered this symptom specific.
  iii. A symptom was considered idiosyncratic if it was extracted from an item that featured only once in a single questionnaire among all the questionnaires analyzed. For instance, the symptom “Hot/cool sensation” is present only in the PSQI in items 5f and 5g “Feel too cold”/“Feel too hot.”

#### 2.3.2. Second Step: Harmonization between Questionnaires

Next, we combined similarly worded or reverse-worded symptoms between questionnaires to ensure that they were not redundant between questionnaires. Symptoms that were judged to be similarly worded or reverse-worded were combined to avoid biasing further analyses. For example, “I have been told that I stop breathing in my sleep” from item 12 of the SDS-CL-25 and “Long pauses between breaths while asleep” from item 11b of the PSQI were lumped into one symptom: “Breath abnormalities observation.” Hence, we did not differentiate between the symptoms “Stop breathing” and “Breathing pause.”

Erring on the side of underestimating heterogeneity where possible as proposed by previous content overlap analysis [23,24,25,26,27,28,29,30], we adopted an original conservative approach based on our previous work of extracting and harmonizing the symptoms in two international classifications of sleep disorders (ICSD-3 and Sleep-Wake disorders section of the DSM-5) [2,3]. The symptoms of the international classifications served as a reference list for combining similarly worded symptoms or reverse-worded symptoms between different questionnaires. This methodology preserves the distinctions made in international classifications between some specific symptoms that should not be combined to improve the consistency of approaches to sleep symptoms [46]. For instance, by comparing prior investigations of symptom content overlap analysis [23,24,25,26,27,28,29,30] and by relying on the sleep literature [12,13], we retained distinct symptoms such as “insomnia initiating” and “insomnia maintaining”, relying on international classifications to facilitate the clinical applicability and international generalizability of our results.

In practice, we harmonized the labels of the symptoms extracted from the questionnaires with those of the two international classifications by applying the following rule: “Is any symptom in any questionnaire similar to any symptom of one of the two international classifications, for all possible combinations?” For instance, the symptom “Stop breathing” extracted from the SDS-CL-25 was harmonized with “Breath abnormalities observation” in the ICSD-3.

When no match was found between any of the extracted symptoms and the list of symptoms in the ICSD-3 or the DSM-5 by the expert panel, we created a new symptom. For instance, the symptom “Dry mouth” was present in the HSDQ, Sleep-50, SDS-CL-25, and SSC but not in the ICSD-3 nor in the DSM-5.

The results of the symptom extraction from each questionnaire and the harmonization of terminology between questionnaires are presented in Supplementary Materials S1.

#### 2.3.3. Third Step: Aggregation of Data in a File

The two previous steps distinguished whether each identified symptom: (i) featured as part of a compound item, (ii) featured as a specific item, (iii) or did not feature in a questionnaire. The third step was the collection of all the symptoms from each questionnaire in a spreadsheet. In this spreadsheet, each line corresponded to a symptom, with the value “1” if the questionnaire included a specific or an idiosyncratic symptom, and the value “2” for compound symptoms. The value “0” was assigned to symptoms not included in a given questionnaire. Each column corresponded to one of the 12 selected questionnaires. Two other columns were for the list of ICSD-3 and DSM-5 sleep symptoms.

We used a network analysis method to organize the extracted symptoms by sleep symptom categories according to our previous systematic analysis of sleep symptoms in the ICSD-3 and the DSM-5 [2,3]. These categories were: sleepiness symptoms, insomnia symptoms, respiratory symptoms, psychiatric symptoms, behavioral symptoms during sleep, motor symptoms, sleep period symptoms, general symptoms, and not otherwise specified. The final spreadsheet is available in Supplementary Materials S1.

### 2.4. Statistical Analysis

We analyzed the symptoms content of self-reported multiple sleep disorder screening questionnaires for adult populations.

#### 2.4.1. Analysis of the Number and Frequency of Sleep Symptoms

We evaluated the number and frequency of symptoms in the 12 questionnaires. We identified the most common and least common symptoms. We evaluated the number and frequency of symptoms in the ICSD-3 and DSM-5 present in the questionnaires and identified symptoms that were not extracted or not in the ICSD-3 and DSM-5. We also identified the questionnaires from which the largest number of symptoms were extracted and counted the number of compound symptoms per questionnaire to evaluate the percentage of items that potentially lacked precision or did not have sufficient symptom specificity per questionnaire. Finally, we evaluated the number of categories represented by the symptoms extracted from each questionnaire and identified the questionnaires that did not evaluate some sleep symptom categories.

#### 2.4.2. Analysis of Content Overlap

We then computed the Jaccard Index (or “Jaccard similarity coefficient”), which is a widely adopted measure of content overlap between different questionnaires [23,24,25,26,27,28,29,30]. The Jaccard Index is a measure of similarity between binary data, ranging from 0 (no overlap among questionnaires) to 1 (complete overlap). It is calculated by the following equation: s/(u1 + u2 + s), with “s” representing the number of symptoms shared by two questionnaires, and “u1” and “u2” representing the number of symptoms that are unique to each one [23]. In line with previous studies that used this type of content analysis [23,24,25,26,27,28,29,30], and considering the absence of well-cited guidelines on the strength or weakness of the Jaccard similarity coefficient, we used the Evans’ Straightforward Statistics for the Behavioral Sciences rule of interpretation for the Jaccard Index [47]: very weak: (0.00–0.19), weak: (0.20–0.39), moderate: (0.40–0.59), strong: (0.60–0.79), and very strong: (0.80–1).

First, the analysis of content overlap was performed for the 12 questionnaires, and the mean overlap for each questionnaire was computed. Second, a pairwise analysis of content overlap was performed between each questionnaire and the ICSD-3 and DSM-5 symptom list.

The correlation between the number of items and the number of symptoms captured by a questionnaire and the mean overlap for the questionnaire was computed to investigate whether the length of the questionnaire played a role in the overlap. The correlation between the percentage of compound sleep symptoms per questionnaire and the mean overlap for the questionnaire was computed to investigate whether the relationship between the proportion of compound symptoms played some role in determining the overlap.

Since all the questionnaires did not explore the same number of sleep symptom categories, we also computed the Jaccard Index for each sleep symptom category to evaluate the symptom content overlap independent of the number of sleep symptom categories explored by the questionnaire.

#### 2.4.3. Data Visualization of the Reproducibility of Content Overlap Results

To facilitate the interpretation of the content overlap, we represented the different symptoms in the questionnaires on an interactive radar plot using the Python package Plotly [48], the symptoms were allocated to angles, while the questionnaires were assigned to different radii.

#### 2.4.4. Availability and Reproducibility of Results

For the sake of total openness and transparency, all our data, results, figures, tables, and the code that generated them are available in a GitHub repository (https://github.com/vincentpmartin/sleep-content-analysis (accessed on 27 December 2022)). Moreover, to foster reproducibility, our Jupyter Notebook has been adapted for other datasets (provided that it is formatted as described in the Method and Supplementary S1) and hosted in a Binder repository (https://mybinder.org/v2/gh/vincentpmartin/sleep-content-analysis/main?labpath=jupyter_notebook_sleep_content_analysis.ipynb (accessed on 27 December 2022)), giving anyone the opportunity to run our notebook online to reproduce our results or to compute the same metrics with custom data.

## 3. Results

### 3.1. Number and Frequency of Sleep Symptoms

**Figure 1:**
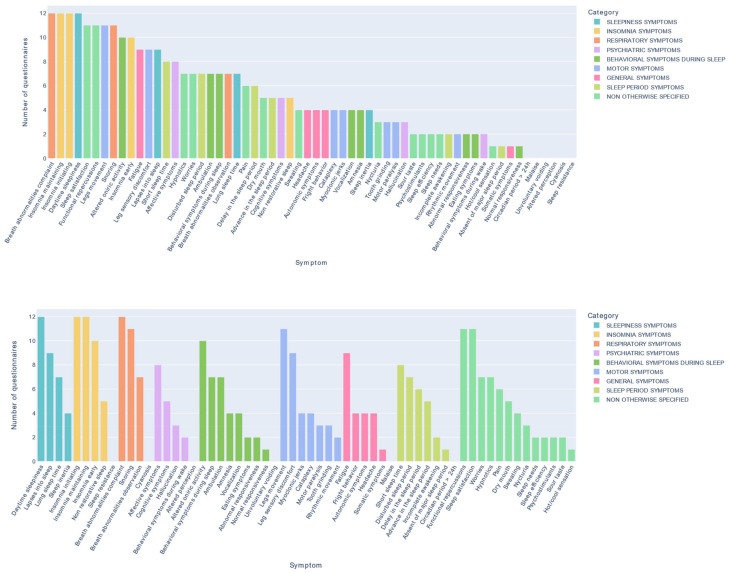
Number of symptoms identified in the 12 selected self-reported multiple sleep disorder screening questionnaires in adults. (**Top**) Organized from the most frequent to the least frequent for all categories of sleep symptoms. (**Bottom**) Organized from the most frequent to the least frequent regarding the category of sleep symptoms as identified previously [2,3,5].

The 12 questionnaires are composed of 469 items that provided 60 different symptoms. Figure 1 shows the number of each symptom.

The number of specific and compound symptoms per questionnaire is presented in Table 1. The number and distribution of sleep symptoms across the sleep symptom categories for each questionnaire are available in Supplementary Materials S2.

The most common symptoms were “Daytime sleepiness,” “Insomnia maintaining,” “Insomnia initiating” and “Breath abnormalities complaint,” which appeared in all 12 questionnaires. The second most frequent symptoms per category were: “Lapses into sleep” in the sleepiness symptoms category; “Insomnia early” in the insomnia symptoms category; “Snoring” in the respiratory symptoms category; “Legs movement” and “Leg sensory discomfort” in the motor symptoms category; “Altered oneiric activity” in the behavioral symptoms during sleep category; “Fatigue” in the general symptoms category; and “Functional repercussion” and “Sleep satisfaction” in the non-otherwise specified category. These symptoms appeared in more than 9/12 questionnaires (75%). Twenty-three extracted symptoms (38.3%) appeared in half of the 12 questionnaires. Four symptoms were idiosyncratic (6.7%), appearing only in one questionnaire, and 17 symptoms appeared in three questionnaires or fewer (28.3%) (Supplementary Materials S1).

Among the 47 sleep symptoms figured in the two international classifications (ICSD-3 and DSM-5), which served as a reference for the present study, 41 (87.2%) were found in the 12 questionnaires. The following six sleep symptoms (12.8%) in the international classifications were not found in the questionnaires: “Malaise,” “Altered perception,” “Involuntary voiding,” “Circadian period > 24 h,” “Cyanosis,” and “Sleep resistance.” Thirteen symptoms extracted from the questionnaires did not correspond to any of the 47 symptoms in the two international classifications. Among them, both “Sleep satisfaction” and “Functional repercussions” were found in 11 of the 12 questionnaires (91.7%). However, “Hot/cool sensation” was an idiosyncratic symptom appearing only in one questionnaire (8.3%): the PSQI. The nine other symptoms not found in the ICSD-3 or DSM-5 had a frequency between 17 and 58%.

The questionnaires containing more than half of the 60 symptoms identified were the SDQ (40/60), the Sleep 50 (34/60), and the ASQ (32/60), which are also long questionnaires: 175 items for the SDQ, 50 for the Sleep 50 and 84 for the ASQ (see Table 1). The ISDI is also a long questionnaire (86 items) but was found to explore only 24/60 symptoms.

The questionnaires with the largest number of compound symptoms were the GSAQ (18/22), the PSQI (12/25), and the HSDQ (12/27) (Table 1).

The BNSQ and the SSC were the questionnaires exploring the smallest number of sleep symptom categories. Psychiatric symptoms were not explored by the SDS-CL-17, the BNSQ, or the OSQ. Behavioral symptoms during sleep were not explored by the SSC or the BNSQ. Motor symptoms were not explored by the BNSQ. The sleep symptom period was not explored by the SSC. The OSQ explored behavioral symptoms during sleep, motor symptoms, and sleep symptom period categories with only one symptom for each. The number of symptoms in each sleep symptom category for each questionnaire is shown in Supplementary Materials S2.

### 3.2. Analysis and Data Visualization of Content Overlap

**Figure 2:**
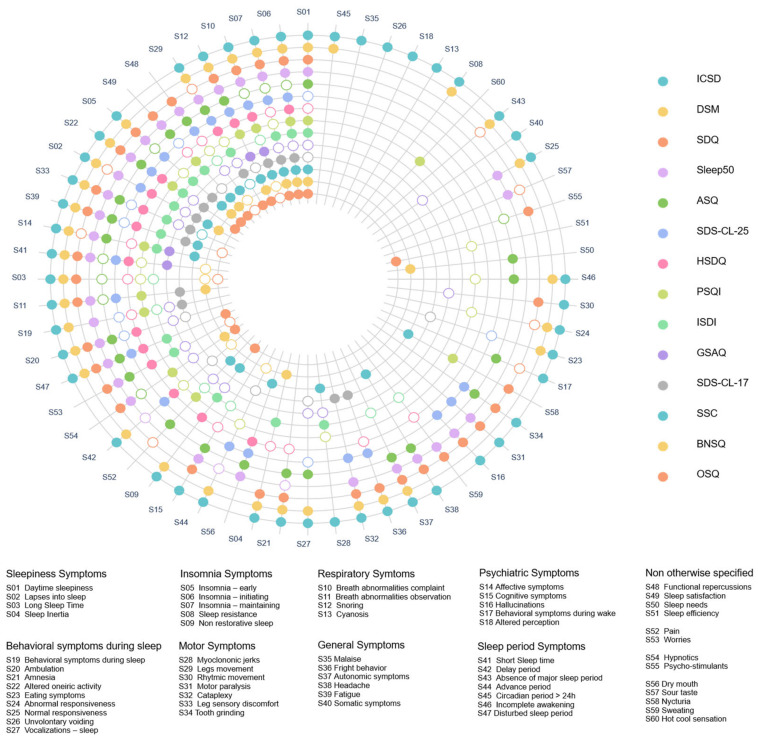
Content overlap of sleep symptoms in the 12 selected self-reported multiple sleep disorder screening questionnaires. Colored circles for a symptom indicate that this is a specific symptom, while empty circles indicate that this is a compound symptom. See also the interactive version of this Figure online: https://chart-studio.plotly.com/~vincent.martin/23/#/ accessed on 27 December 2022. ICSD: International Classification of Sleep Disorders, DSM: Diagnostic and Statistical Manual of Mental Disorders, SDQ: Sleep Disorder Questionnaire, ASQ: Auckland Sleep Questionnaire, HSDQ: Holland Sleep Disorders Questionnaire, SDS-CL-25: Sleep Disorder Symptom Checklist 25, PSQI: Pittsburgh Sleep Quality Index, ISDI: Iowa Sleep Disturbances Inventory, GSAQ: Global Sleep Assessment Questionnaire, SDS-CL-17: Sleep Disorder Symptom Checklist 17, SSC: Sleep Symptom Checklist, BNSQ: Basic Nordic Sleep Questionnaire, OSQ: Oviedo Sleep Questionnaire.

Figure 2 shows the analysis of the content overlap of sleep symptoms in the 12 questionnaires.

**Figure 3:**
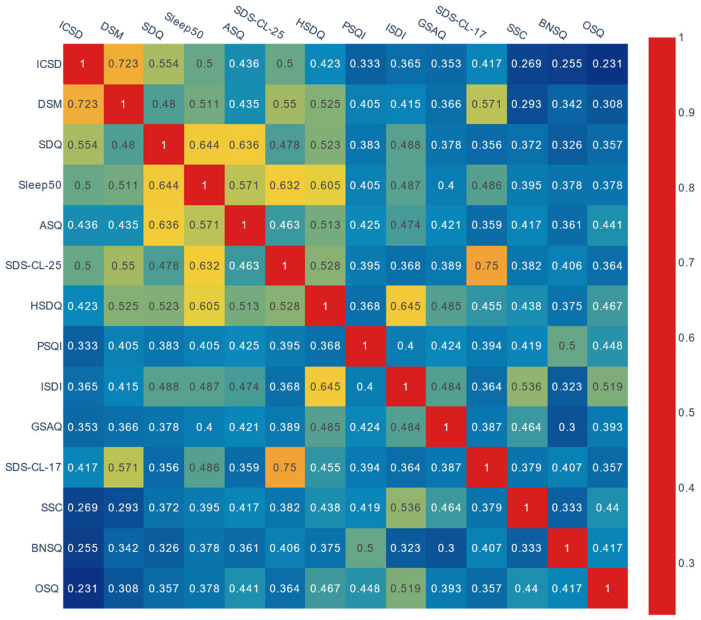
Jaccard Index overlap of item content of the 12 self-reported multiple sleep disorder screening questionnaires for each pair of questionnaires. ICSD: International Classification of Sleep Disorders, DSM: Diagnostic and Statistical Manual of Mental Disorders, SDQ: Sleep Disorder Questionnaire, ASQ: Auckland Sleep Questionnaire, HSDQ: Holland Sleep Disorders Questionnaire, SDS-CL-25: Sleep Disorder Symptom Checklist 25, PSQI: Pittsburgh Sleep Quality Index, ISDI: Iowa Sleep Disturbances Inventory, GSAQ: Global Sleep Assessment Questionnaire, SDS-CL-17: Sleep Disorder Symptom Checklist 17, SSC: Sleep Symptom Checklist, BNSQ: Basic Nordic Sleep Questionnaire, OSQ: Oviedo Sleep Questionnaire.

Average Jaccard indexes per questionnaire are presented in Table 1. The pairwise analysis of content overlap performed between each questionnaire and between each questionnaire and the ICSD-3 and DSM-5 symptom list is presented in Figure 3.

The mean overlap between the questionnaires evaluated with the Jaccard Index was 0.44, indicating a moderate similarity [23,47].

The questionnaires that overlapped the most with the ICSD-3 and the DSM-5 symptoms were the SDS-CL-25, the SDQ, and the Sleep 50 (Figure 3). The questionnaires that overlapped the least with the ICSD-3 and the DSM-5 symptoms were the OSQ, the SSC, and the BNSQ.

As shown in Table 1, the questionnaires with the highest average Jaccard indexes in descending order were the Sleep 50 (0.49), the HSDQ (0.49), the SDS-CL-25 (0.47), the ISDI (0.46), the ASQ (0.46), and the SDQ (0.45). The three latter questionnaires were also those with the highest number of items (respectively, 86, 84, and 175). The BNSQ and the GSAQ had the lowest average Jaccard indexes (respectively, 0.37 and 0.41), with the GSAQ having the lowest number of items (*n* = 11). The correlation between the number of items and the mean overlap was 0.645 (*p* = 0.02). The correlation between the number of extracted symptoms and the mean overlap was 0.658 (*p* = 0.02). The correlation between the percentage of compound sleep symptoms per questionnaire, with the mean overlap for the questionnaire, was not significant.

The SDS-CL-17 and the SDS-CL-25 had the highest pairwise overlap at 0.75, followed by the ISDI with the HSDQ (0.65) and the Sleep 50, and the SDQ (0.64). The BNSQ and the GSAQ had the lowest pairwise overlap at 0.30 (Figure 3).

The mean overlap among all the questionnaires was the highest for insomnia symptoms (0.79), respiratory symptoms (0.76), and sleepiness symptoms (0.61), while it was less than 0.5 for the other sleep symptom categories (Supplementary Materials S2).

## 4. Discussion

This paper is, to our knowledge, the first to apply the method developed by Fried to perform a systematic analysis of sleep symptoms in a set of self-reported multiple sleep disorder screening questionnaires for adult populations [22,23] and to offer a handy tool to perform content overlap analysis online with custom data (https://mybinder.org/v2/gh/vincentpmartin/sleep-content-analysis/main?labpath=jupyter_notebook_sleep_content_analysis.ipynb (accessed on 27 December 2022)). Our analysis offers a broad appraisal of a set of symptoms important to sleep medicine.

Firstly, we found that “insomnia initiating,” “insomnia maintaining”, and “daytime sleepiness” symptoms were investigated by all the questionnaires, confirming the central role of these symptoms in sleep medicine since the Diagnostic Classification of Sleep and Arousal Disorders (DCSAD) was proposed in 1979 [49]. The major dichotomy of the DCSAD was indeed between disorders of initiating and maintaining wakefulness (DIMS) and disorders of excessive somnolence (DOES). Our results are in line with this historical perspective, which can be seen as one of the first attempts to seriously consider these two kinds of sleep symptoms as having a central role in the structure of the classification of sleep disorders. Moreover, we found that respiratory symptoms were also investigated by almost all the questionnaires. “Breath abnormalities complaint” was found in all the questionnaires, in various wordings such as “I wake up choking or gasping for air” in the SDS-CL-25, “Cannot breathe comfortably” in the PSQI, or “I wake suddenly gasping for breath, unable to breath” in the SDQ. “Snoring” was found in all the questionnaires except the ISDI. The prominent role of respiratory symptoms can be explained by the place occupied by obstructive sleep apnea syndrome (OSAS) in the development of sleep medicine [7,50]. Interestingly, these three categories of symptoms (insomnia, somnolence, and respiratory symptoms) exhibited the highest averaged Jaccard Index, suggesting a strong content overlap between the questionnaires for these symptoms. This confirms the specific attention from sleep medicine to these symptoms in sleep disorder questionnaires.

Secondly, we found that the overlap between the sleep symptoms extracted from the self-reported multiple sleep disorder screening questionnaires for adult populations was moderate. One explanation could be that each questionnaire explores a different number of main sleep disorder categories, from 3 to 6, as discussed by Klingman et al. 2017 [22]. Not exploring the same set of main sleep disorder categories can lead to a different set of sleep symptoms between questionnaires, thus reducing the mean overlap content. In line with this explanation, we found that, on average, questionnaires that explore the greatest number of symptoms with the greatest number of investigated sleep symptom categories exhibit the highest mean overlap. Indeed, unlike Fried, we found almost significant relationships between the number of symptoms probed by a questionnaire and the mean overlap, suggesting that questionnaire length is involved in determining overlap [23]. Thus, heterogeneity might be partly attributable to the comparison of questionnaires that were developed for a different set of sleep disorders.

Nevertheless, to avoid the results being biased by the number of sleep disorders screened by the questionnaires, we evaluated the Jaccard index specifically for each category of sleep symptoms. Interestingly, we found a high mean overlap for the sleep symptom categories of insomnia, sleepiness, and respiratory symptoms, while the mean overlap in the categories of psychiatric symptoms, behavioral symptoms during sleep, motor symptoms, sleep period symptoms, and general symptoms was low or moderate. These observations suggest another hypothesis that could explain the moderate average overlap between the symptoms extracted from the questionnaires: a lack of consensus on the list and terminology of the sleep symptoms that are not the central symptoms that were used to structure and develop the sleep disorders classification [2,3]. Heterogeneity might be partly attributable to the comparison of questionnaires that were developed with different expert advice. Indeed, the content of each questionnaire is based on a combination of empirical data, expert opinion, and consensus on specific expert advice. Due to the lack of a specific and consensual set of sleep symptoms in the different versions of the ICSD or the DSM [2,3,5,20], each questionnaire was developed on the basis of the interpretation of diagnostic criteria, from which it could be difficult to identify ‘symptoms’. Moreover, it is interesting to note that this content analysis demonstrates a strong overlap with the sleep symptoms considered central by the sleep community, as shown in our previous analysis [2,3]. Indeed, it seems that the more peripheral the sleep symptoms are, using the network analysis method, developed by Borsboom [31], the less there is an overlap between them, using the content overlap analysis developed by Fried [23]. Such a relationship should be confirmed and better quantified, but the intertwining of these two kinds of analyses seems interesting to better investigate how the sleep community considers sleep symptoms.

Thirdly, we propose to complement our content analyses with the comprehensiveness, brevity, and psychometric quality analyses made by Klingman et al. [22].

The HSDQ [35], the Sleep 50 [38], and the SDS-CL-25 [42] questionnaires displayed the highest mean overlap with other questionnaires for sleep symptoms. Nevertheless, the HSDQ contains a high number of compound symptoms (Table 1), which might reduce the practicability and reliability of such a questionnaire [22]. The Sleep 50 has fewer compound symptoms than the HSDQ and is more concise and precise in the phrasing of items [22], resulting in a good match with the symptom terms used to perform the content analysis. Moreover, the Sleep 50 exhibits a relatively high mean content overlap with the symptoms of the ICSD-3 and DSM-5 international classifications. Due to its relatively high overlap, it might be a good choice for those wishing to perform systematic sleep symptom analysis [38].

Although it does not screen for some sleep disorders [42], we found that the GSAQ [37], which has been considered suitable to screen for sleep disorders in terms of the comprehensiveness, brevity, and psychometric quality analyses made by Klingman et al. [22], displayed a lower mean overlap with the other questionnaires for sleep symptoms and a high number of compound symptoms. We thus confirm the added value of the work of Klingman et al. in developing the SDS-CL-25, which fills the gaps left by the GSAQ [42]. Moreover, we found that the SDS-CL-25 has a higher mean content overlap than the GSAQ, exhibits a higher mean content overlap with the symptoms of the ICSD-3 and DSM-5 international classifications, and probes a lower number of compound symptoms. The SDS-CL-25 has fewer items than the Sleep 50, yet has a similar mean overlap and is more concise and precise in its phrasing. It is thus a good choice for those seeking an explorative evaluation of sleep symptoms, allowing a broad screening of sleep disorders in a practicable, reliable, and valid manner while respecting the international classifications [42]. Our results also suggest that the SDS-CL-25 is more efficient than the SDS-CL-17, which has a lower mean overlap despite having a higher pairwise overlap [22].

The ISDI [36], the SDQ [34], and the ASQ [32] displayed satisfactory mean overlap with other questionnaires for sleep symptoms and relatively high pairwise overlap with the HSDQ [35], the Sleep 50 [38], and the SDS-CL-25 [42]. However, they contain a large number of items, with poor brevity and standardization in the phrasing. For this reason, as suggested by Klingman, they cannot be considered a good choice for screening sleep disorders, despite relatively satisfactory sleep content analysis results [22].

The BNSQ [41] and the OSQ [40], which were not included in the systematic review of Klingman et al. [22], displayed a low mean overlap with the other questionnaires for sleep symptoms. This lack of overlap can be explained by the fact that neither of them explores many sleep symptom categories. They are therefore suitable neither for systematic sleep symptom analysis nor for screening sleep disorders. Interestingly, while the PSQI is the most widely used questionnaire in research on adults [39,51], its content overlap is relatively low. In particular, it does not overlap well with the symptoms of the international classifications ICSD-3 and DSM-5. One explanation for its wide use in everyday clinical practice could be that it was the first self-reported multiple sleep disorder screening questionnaire for adult populations in the 1990s. More recent questionnaires, such as the SDS-CL-25 [42], seem to have adjusted and modified the list of symptoms to keep abreast of accruing knowledge in sleep medicine and the diagnostic criteria of international classifications.

This study has some limitations. First, we selected the questionnaires without performing a systematic analysis. However, we used the results of the systematic analysis of Klingman et al., which they performed recently in 2017 [22]. Moreover, we used a replicable selection method previously discussed in studies of content analysis [23,24,25,26,27,28,29,30]. More generally, our results cannot be extended to all questionnaires assessing sleep disorders, given that we only analyzed self-reported questionnaires and excluded structured questionnaires for clinical interviews, which also warrant symptom content analysis [20]. We previously analyzed the reliability of these questionnaires for clinical interviews and overlap content analysis should be performed in further studies [20].

The second limitation is related to the absence of content overlap analysis for specific sleep disorders. Indeed, we focused on self-reported multiple sleep disorder screening questionnaires, which are multidimensional. In this way, future studies will be needed to analyze sleep constructs through content overlap analysis of self-reported screening questionnaires specific to a sleep disorder (e.g., insomnia disorder, hypersomnia disorder, obstructive sleep apnea syndrome, or circadian sleep–wake disorders).

The third limitation is to lump in our analysis all the clinical manifestations of items related to “Functional repercussions”, which we have considered “sleep harms” in our previous ICSD-3 diagnostic criteria analysis [5]. This can lead to a confusion between the notions of “sleep symptoms” and “sleep harms”, which tend to be both present indistinctly in the self-reported multiple sleep disorder screening questionnaires investigated. Further studies are thus necessary to better investigate the construct of sleep functional repercussions and sleep harms by content overlap analysis.

The fourth limitation is related to symptom extraction and harmonization, steps that are subjective by nature. We assumed that lumping and splitting, as well as rewording, would have produced slightly different results if performed by another research group. Nevertheless, we used a double-blind approach based on a panel of medical examiners outside the sleep community, on the one hand, and on sleep experts on the other hand, to ensure the reliability of our procedure according to methodologies already proven in large epidemiological samples with independent examiners [43,44,45]. Moreover, we constrained symptom extraction and harmonization by the previously published set of symptoms extracted from two international classifications (ICSD-3 and DSM-5) [2,3], in order to ensure coherence and external validity in the scientific literature [5].

The final limitation is related to the interpretation of the Jaccard coefficients, given the absence of established rules for their interpretation. Nevertheless, we used the methods and interpretation performed in previous content overlap analyses [23,24,25,26,27,28,29,30].

## 5. Conclusions

Given the heterogeneity observed in self-reported multiple sleep disorder screening questionnaires for the adult population, especially for symptoms other than insomnia, somnolence, and sleep-related breathing symptoms, a consensual set of sleep symptoms should be determined in sleep medicine in order to enhance the practicability, reliability, and validity of sleep disorder diagnoses. Even though the SDS-CL-25 offers a broad screening of sleep disorders, future systematic sleep symptom analysis requires new tools. Two complementary solutions could be combined to standardize the contents of sleep symptoms. First, there should be an expert consensus on sleep symptoms in sleep medicine leading to a consensual terminology, as recently proposed for hypersomnolence symptoms [16]. While the symptoms and the terminology to describe them could be evolutive, the objective would be to avoid unnecessary variability in how symptoms are defined in the sleep literature [5,6]. Second, a sleep item data hub should be set up, and specific empirical analysis should be encouraged. In addition to expert assessment, a process for collecting item sets to establish pools of items would be useful for identifying the most important sets of sleep symptoms (and sleep harms) in clinical sleep medicine and sleep health evaluation. Such a process could rely on existing questionnaires, item standardization, content validity testing, and refinement via item response theory (IRT) and other psychometric methods such as network analysis. A promising approach is the Patient-Reported Outcomes Measurement Information System (PROMIS) applied recently to the sleep medicine field [21], which provides scope for a fine-tuned patient-centered approach to symptoms and harms. In conclusion, it is by paying careful attention to the content of sleep symptoms that the sleep medicine community will be able to develop standardized self-reported multiple sleep disorder screening questionnaires based on the ICSD and DSM diagnostic criteria and on empirical research in sleep medicine in order to assess sleep symptoms more systematically.

## Figures and Tables

**Table 1:** Average Jaccard index, number of specific and compound symptoms that appear across self-reported multiple sleep disorder screening questionnaires. ICSD: International Classification of Sleep Disorders, DSM: Diagnostic and Statistical Manual of Mental Disorders, SDQ: Sleep Disorder Questionnaire, ASQ: Auckland Sleep Questionnaire, HSDQ: Holland Sleep Disorders Questionnaire, SDS-CL-25: Sleep Disorder Symptom Checklist 25, PSQI: Pittsburgh Sleep Quality Index, ISDI: Iowa Sleep Disturbances Inventory, GSAQ: Global Sleep Assessment Questionnaire, SDS-CL-17: Sleep Disorder Symptom Checklist 17, SSC: Sleep Symptom Checklist, BNSQ: Basic Nordic Sleep Questionnaire, OSQ: Oviedo Sleep Questionnaire.

	Average Jaccard Index	Items	Specific Symptoms	Compound Symptoms	Total Number of Symptoms
SDQ	0.449	175	32	8	40
Sleep50	0.498	50	31	3	34
ASQ	0.462	84	23	9	32
SDS-CL-25	0.469	25	20	8	28
HSDQ	0.491	32	15	12	27
PSQI	0.415	24	13	12	25
ISDI	0.462	86	16	8	23
GSAQ	0.411	11	4	18	22
SDS-CL-17	0.427	17	13	8	21
SSC	0.416	22	17	2	19
BNSQ	0.375	22	11	6	17
OSQ	0.416	19	12	5	17

## Data Availability

Not applicable.

## Supplementary Materials

The following supporting information can be downloaded at: https://www.mdpi.com/article/10.3390/jcm12030852/s1, Supplementary Material 1: Item extraction and selection for each questionnaire, and symptom extraction and terminology harmonization between questionnaires. Supplementary Material 2: Analysis notebook containing all the results and interactive figures. All our code, data, figures and tables can be found at: https://github.com/vincentpmartin/sleep-content-analysis (accessed on 27 December 2022); A binder repository to reproduce our results online with custom data is available at: https://mybinder.org/v2/gh/vincentpmartin/sleep-content-analysis/main?labpath=jupyter_notebook_sleep_content_analysis.ipynb (accessed on 27 December 2022).
