# Supplementary material for: Systematic Item Content and Overlap Analysis of Self-Reported Multiple Sleep Disorder Screening Questionnaires in Adults
Source: J Clin Med. 2023 Jan 20;12(3):852. doi: 10.3390/jcm12030852 (PMC9918039; doi:10.3390/jcm12030852)
Supplement: Supplementary file 1 [file jcm-12-00852-s001.zip › Supplementary Material S2.html]

jupyter\_notebook\_sleep\_content\_analysis


# Sleep Content Analysis (or Any-Other-Construct Content Analysis)¶

## ⚠️ PLEASE READ BEFORE DOING ANYTHING ⚠️¶

Welcome to this online coding environment !
You are currently running a *Jupyter notebook* that we hope to be usefull for content analysis of questionnaires.

> Each cell can be ran individually (pressing the "Play" ▶ button or pressing CTRL+ENTER in the each cell). In order to make everything run smoothly, it is **strongly** recommended to run them in order.

> \***RECOMMANDED:**\* You can also run all the cells at the same time : "Run" → "Run All Cells".  
> ⚠️ If you choose this option and want to analyse your own data, we suggest that you configure correctly the `experiment`, `import_file` and `references` variables before running all the cells once, then adjust the `max_radius` variable of Figure 4.

At the end of the execution, you will have the opportunity to save all yours results in an HTML file, executing `"File" → "Save and Export Notebook as" → "HTML"`

If you have chosen to run every cell individually, please begin by running the following cell that imports all the required package for our code to work correctly ↓

In [37]:

```
%%capture
import pandas as pd
import numpy as np 
from sklearn.metrics import jaccard_score
import plotly.express as px
import plotly.graph_objects as go
from sklearn.preprocessing import normalize
from scipy.stats import spearmanr

#Required package to download the image
!wget https://github.com/plotly/orca/releases/download/v1.2.1/orca-1.2.1-x86_64.AppImage -O /usr/local/bin/orca
!chmod +x /usr/local/bin/orca
!apt-get install xvfb libgtk2.0-0 libgconf-2-4
```

---

# Data import¶

## Correct data formatting¶

By default, this repository is configured to reproduce the results of the following paper (option `experiment = "GauldMartin2022"`):

- Gauld C, Martin VP, Richaud A, Bailleul S, Lucie V, Perromat JL, et al. Systematic Item Content and Overlap Analysis of Self-reported Multiple Sleep Disorders Screening Questionnaires in Adults. *Journal of Clinical Medicine*. 2022 (Under review)

We also implemented the analysis for the replication of the seminal paper by E. Fried (option `experiment = "Fried2017"`) :

- Fried EI. The 52 symptoms of major depression: Lack of content overlap among seven common depression scales. *Journal of Affective Disorders*. 2017 Jan;208:191–7.

However, we have coded it to be adaptable to any other dataset (`experiment = "Custom"`), formatted the following way :

- the three first columns must be the category (named "Category" in our example), the abbreviation for the symptom (as shown in the Figure, named "Ab" in our example) and the name of the symptom ("Symptom" in our example);
- the other columns are the different questionnaires, while the line are the different symptoms.
- For each questionnaire, the symptoms are coded the following way:
  - 0: The symptom is absent from this questionnaire
  - 1: The symptom is specific in this questionnaire (i.e. the symptom has bene identified in an item mentionning only one symptom)
  - 2: The symptom is componed in this questionnaire (i.e. the symptom has bene identified in an item mentionning at least two symptom)

⚠️ If you do not have categories, just put an empty columns as first column ⚠️

## Importing data by uploading an excel file¶

To import your own file in the binder online folder :

> - Open the online folder pressing the "folder" icon on the left 📁
> - Click on the "Upload a file" button ⭱

Now adapt the `experiment` and `import_file` to your experiment !

In [38]:

```
# uncomment the analysis you want to run
#experiment = "Fried2017"
experiment = "GauldMartin2022"
#experiment = "Custom"

if experiment == "Fried2017" : 
    df = pd.read_excel("./data/fried2017_reformatted.xlsx") # reproduction of the seminal paper of Fried et al. https://doi.org/10.1016/j.jad.2016.10.019
elif experiment=="GauldMartin2022" : 
    df = pd.read_excel("./data/database_symptoms_sleep_content_analysis.xlsx")
else :
    import_file = "./your_excel_file.xlsx"
    df = pd.read_excel(import_file)
    
df.rename(columns={df.columns[0]: "Category", df.columns[1]: "Ab", df.columns[2]: "Symptom"}, inplace=True) #replacing the name of the three first columns !
df.sort_values(by="Ab",inplace = True) # sort the dataset by abbreviation
df.head() # print the 5 first rows
```

Out[38]:

|  | Category | Ab | Symptom | SDQ | ICSD | ASQ | GSAQ | HSDQ | PSQI | Sleep50 | DSM | SDS-CL-25 | ISDI | SDS-CL-17 | BNSQ | OSQ | SSC |
| --- | --- | --- | --- | --- | --- | --- | --- | --- | --- | --- | --- | --- | --- | --- | --- | --- | --- |
| 0 | SLEEPINESS SYMPTOMS | S01 | Daytime sleepiness | 1 | 1 | 1 | 2 | 2 | 1 | 1 | 1 | 2 | 1 | 2 | 1 | 1 | 1 |
| 1 | SLEEPINESS SYMPTOMS | S02 | Lapses into sleep | 2 | 1 | 0 | 2 | 1 | 0 | 1 | 1 | 1 | 1 | 1 | 1 | 0 | 1 |
| 2 | SLEEPINESS SYMPTOMS | S03 | Long sleep time | 1 | 1 | 2 | 0 | 2 | 2 | 0 | 1 | 0 | 2 | 0 | 2 | 2 | 0 |
| 3 | SLEEPINESS SYMPTOMS | S04 | Sleep inertia | 1 | 1 | 0 | 0 | 2 | 0 | 0 | 1 | 0 | 1 | 0 | 1 | 0 | 0 |
| 4 | INSOMNIA SYMPTOMS | S05 | Insomnia early | 1 | 1 | 2 | 0 | 0 | 2 | 1 | 1 | 1 | 1 | 1 | 1 | 1 | 1 |

## Reference classifications¶

In our paper, we compare the symptoms of the questionnaires with two references classification (ICSD and DSM). If you have reference columns that you want to compare with but you do not want to compute metrics on, please put them in this table. Otherwise, just let this list empty [ ].

⚠️ the name of the references should match PERFECTLY the name of the columns they are in (including uppercases and lowercases, or spaces)

In [39]:

```
if experiment == "Fried2017" :
    references = []
elif experiment == "GauldMartin2022": 
    references = ['ICSD', 'DSM']
else : 
    references = ['ref1', 'ref2', '...']
header = ['Category', 'Ab', 'Symptom']+references
```

# Ordering questionnaires and symptoms¶

First, the questionnaires are classified from having the higher number of symptoms to the lowest.

In [40]:

```
sums = (df.drop(header,axis = 1)>=1).sum(axis = 0) # sum of the number of symptom by questionnaire
col = list(sums.sort_values(ascending=False).index.to_numpy()) #we create the list of columns
col = header + col
# we apply the order of columns to the dataset
df = df.loc[:, col]
df.head()
```

Out[40]:

|  | Category | Ab | Symptom | ICSD | DSM | SDQ | Sleep50 | ASQ | SDS-CL-25 | HSDQ | PSQI | ISDI | GSAQ | SDS-CL-17 | SSC | BNSQ | OSQ |
| --- | --- | --- | --- | --- | --- | --- | --- | --- | --- | --- | --- | --- | --- | --- | --- | --- | --- |
| 0 | SLEEPINESS SYMPTOMS | S01 | Daytime sleepiness | 1 | 1 | 1 | 1 | 1 | 2 | 2 | 1 | 1 | 2 | 2 | 1 | 1 | 1 |
| 1 | SLEEPINESS SYMPTOMS | S02 | Lapses into sleep | 1 | 1 | 2 | 1 | 0 | 1 | 1 | 0 | 1 | 2 | 1 | 1 | 1 | 0 |
| 2 | SLEEPINESS SYMPTOMS | S03 | Long sleep time | 1 | 1 | 1 | 0 | 2 | 0 | 2 | 2 | 2 | 0 | 0 | 0 | 2 | 2 |
| 3 | SLEEPINESS SYMPTOMS | S04 | Sleep inertia | 1 | 1 | 1 | 0 | 0 | 0 | 2 | 0 | 1 | 0 | 0 | 0 | 1 | 0 |
| 4 | INSOMNIA SYMPTOMS | S05 | Insomnia early | 1 | 1 | 1 | 1 | 2 | 1 | 0 | 2 | 1 | 0 | 1 | 1 | 1 | 1 |

In [41]:

```
#order of symptoms: most common first (independtly from 'specific' or 'compound').
df['sum_symptoms'] = (df.drop(header,axis = 1)>=1).sum(axis = 1)
df.sort_values(by=['sum_symptoms','Ab'], ascending = [False,True], inplace = True)
df.head()
```

Out[41]:

|  | Category | Ab | Symptom | ICSD | DSM | SDQ | Sleep50 | ASQ | SDS-CL-25 | HSDQ | PSQI | ISDI | GSAQ | SDS-CL-17 | SSC | BNSQ | OSQ | sum\_symptoms |
| --- | --- | --- | --- | --- | --- | --- | --- | --- | --- | --- | --- | --- | --- | --- | --- | --- | --- | --- |
| 0 | SLEEPINESS SYMPTOMS | S01 | Daytime sleepiness | 1 | 1 | 1 | 1 | 1 | 2 | 2 | 1 | 1 | 2 | 2 | 1 | 1 | 1 | 12 |
| 5 | INSOMNIA SYMPTOMS | S06 | Insomnia initiating | 1 | 1 | 1 | 1 | 2 | 1 | 1 | 1 | 1 | 2 | 1 | 1 | 1 | 1 | 12 |
| 6 | INSOMNIA SYMPTOMS | S07 | Insomnia maintaining | 1 | 1 | 1 | 1 | 2 | 1 | 2 | 2 | 1 | 2 | 1 | 1 | 1 | 1 | 12 |
| 9 | RESPIRATORY SYMPTOMS | S10 | Breath abnormalities complaint | 1 | 1 | 1 | 1 | 2 | 1 | 1 | 1 | 2 | 1 | 1 | 1 | 2 | 2 | 12 |
| 11 | RESPIRATORY SYMPTOMS | S12 | Snoring | 1 | 1 | 1 | 1 | 1 | 1 | 1 | 1 | 0 | 1 | 2 | 1 | 1 | 1 | 11 |

---

# 1. Analysis of the number and frequency of symptoms¶

In a first step, we analyse the frequency of the symptoms.

## Histogram of number of symptoms¶

### Sorted by number of occurences¶

In [42]:

```
df_questionnaires = df.drop(references, axis = 1)
if df.shape[0] != df['Category'].isnull().sum() : 
    color = 'Category'
else : 
    color = "sum_symptoms"
fig = px.bar(df_questionnaires.sort_values(by=['sum_symptoms','Ab'],ascending = [False,True]),
             x='Symptom',
             y='sum_symptoms',
             color=color,
             labels={'sum_symptoms':'Number of questionnaires'},
             color_discrete_sequence =  px.colors.qualitative.Pastel, ## more color palettes available here : https://plotly.com/python/builtin-colors
             category_orders = {'Category':df_questionnaires.sort_values(by='Ab').Category.unique()}
             )
fig.update_layout(xaxis_tickangle=-60,autosize=False, width=1500, height=600, 
                  #xaxis={'categoryorder': 'total descending'}
                  xaxis={'categoryorder':'array', 'categoryarray':df_questionnaires.Symptom.unique()},

                  )
fig.write_image("figure1_histogram_1.pdf")
fig.show()
```

The figure has been save in the online folder (📁 symbol on the left) under the name figure1\_histogram\_1.pdf.   
You can change the name and the format of the file changing the name in the `fig.write_image()` function.
  
⚠️ If you need it, save the figure on your local computer : these online file will be deleted as soon as you quit this page!

### Sorted by category¶

In [43]:

```
if df.shape[0] != df['Category'].isnull().sum() : 
    color = 'Category'
else : 
    color = "sum_symptoms"
fig = px.bar(df_questionnaires.sort_values(by=['sum_symptoms','Ab'],ascending = [False,True]),
             x='Symptom',
             y='sum_symptoms',
             color=color,
             labels={'sum_symptoms':'Number of questionnaires'},
             color_discrete_sequence =  px.colors.qualitative.Pastel, # more color palettes available here : https://plotly.com/python/builtin-colors
             category_orders = {'Category':df_questionnaires.sort_values(by='Ab').Category.unique()}
            )
fig.update_layout(xaxis_tickangle=-60,autosize=False, width=1500, height=600)
fig.write_image("figure2_histogram_2.pdf")
fig.show()
```

The figure has been save in the online folder (📁 symbol on the left) under the name figure2\_histogram\_2.pdf.   
You can change the name and the format of the file changing the name in the `fig.write_image()` function.
  
⚠️ If you need it, save the figure on your local computer : these online file will be deleted as soon as you quit this page!

## Number of symptoms by questionnaire¶

In [44]:

```
sympt_per_questionnaire = pd.DataFrame(np.zeros((df.shape[1]-4,3)), index = df.iloc[:,3:-1].columns, columns = ['Specific symptoms', 'Compound symptoms', 'Total'])
sympt_per_questionnaire['Specific symptoms'] = (df.iloc[:,3:-1]==1).sum(axis = 0)
sympt_per_questionnaire['Compound symptoms'] = (df.iloc[:,3:-1]==2).sum(axis = 0)
sympt_per_questionnaire['Total'] = (df.iloc[:,3:-1]>=1).sum(axis = 0)
sympt_per_questionnaire.to_excel("table1_symptomes_per_questionnaire.xlsx")

display(sympt_per_questionnaire)
```

|  | Specific symptoms | Compound symptoms | Total |
| --- | --- | --- | --- |
| ICSD | 47 | 0 | 47 |
| DSM | 34 | 0 | 34 |
| SDQ | 32 | 8 | 40 |
| Sleep50 | 31 | 3 | 34 |
| ASQ | 23 | 9 | 32 |
| SDS-CL-25 | 20 | 8 | 28 |
| HSDQ | 15 | 12 | 27 |
| PSQI | 13 | 12 | 25 |
| ISDI | 16 | 8 | 24 |
| GSAQ | 4 | 18 | 22 |
| SDS-CL-17 | 13 | 8 | 21 |
| SSC | 17 | 2 | 19 |
| BNSQ | 11 | 6 | 17 |
| OSQ | 12 | 5 | 17 |

The table has been save in the online folder (📁 symbol on the left) under the name table1\_symptoms\_per\_questionnaire.xlsx.   
You can change the name and the format of the file changing the name in the `sympt_per_questionnaire.to_excel()` function.
  
⚠️ If you need it, save the excel file on your local computer : these online file will be deleted as soon as you quit this page!

## Symptoms that are in classifications but not in questionnaires¶

In [45]:

```
if len(references) > 0 :
  for ref in references: 
    print("---", ref, "---")
    for symptom in df.Symptom.unique() : 
      if (int(df.loc[df.Symptom == symptom,ref]) == 1 and int(df.loc[df.Symptom == symptom, 'sum_symptoms'])==0) : 
        print(symptom)
else : 
    print("References list is empty")
```

```
--- ICSD ---
Sleep resistance
Cyanosis
Altered perception
Unvoluntary voiding
Malaise
Circadian period > 24h
--- DSM ---
Sleep resistance
Circadian period > 24h
```

## Number of symptoms in each category for each questionnaire¶

In [46]:

```
# number of categories/questionnaire
if df.shape[0] != df['Category'].isnull().sum() : 
  cat_per_questionnaire = pd.DataFrame(np.zeros((df.shape[1]-4,len(df.Category.unique()))), index = df.iloc[:,3:-1].columns, columns = df.sort_values(by="Ab").Category.unique())
  for category in df.Category.unique():
    cat_per_questionnaire.loc[:,category] = (df[df.Category==category].iloc[:,3:-1]>=1).sum(axis = 0)
  display(cat_per_questionnaire.T)
  cat_per_questionnaire.T.to_excel("table2_categorie_per_questionnaire.xlsx")
else : 
  print('No category in this dataframe !')
```

|  | ICSD | DSM | SDQ | Sleep50 | ASQ | SDS-CL-25 | HSDQ | PSQI | ISDI | GSAQ | SDS-CL-17 | SSC | BNSQ | OSQ |
| --- | --- | --- | --- | --- | --- | --- | --- | --- | --- | --- | --- | --- | --- | --- |
| SLEEPINESS SYMPTOMS | 4 | 4 | 4 | 2 | 2 | 2 | 4 | 2 | 4 | 2 | 2 | 2 | 4 | 2 |
| INSOMNIA SYMPTOMS | 5 | 5 | 3 | 3 | 3 | 3 | 3 | 3 | 4 | 3 | 3 | 4 | 3 | 4 |
| RESPIRATORY SYMPTOMS | 4 | 3 | 3 | 3 | 3 | 3 | 2 | 3 | 1 | 2 | 3 | 2 | 3 | 2 |
| PSYCHIATRIC SYMPTOMS | 5 | 1 | 4 | 3 | 3 | 1 | 2 | 1 | 2 | 1 | 0 | 1 | 0 | 0 |
| BEHAVIORAL SYMPTOMS DURING SLEEP | 9 | 8 | 4 | 5 | 4 | 5 | 4 | 3 | 2 | 4 | 5 | 0 | 0 | 1 |
| MOTOR SYMPTOMS | 7 | 3 | 6 | 4 | 3 | 5 | 2 | 2 | 3 | 4 | 3 | 3 | 0 | 1 |
| GENERAL SYMPTOMS | 6 | 3 | 4 | 3 | 3 | 2 | 2 | 0 | 2 | 1 | 2 | 2 | 0 | 1 |
| SLEEP PERIOD SYMPTOMS | 7 | 7 | 3 | 4 | 3 | 4 | 3 | 4 | 1 | 1 | 2 | 0 | 3 | 1 |
| NON OTHERWISE SPECIFIED | 0 | 0 | 9 | 7 | 8 | 3 | 5 | 7 | 5 | 4 | 1 | 5 | 4 | 5 |

The table has been save in the online folder (📁 symbol on the left) under the name table2\_categorie\_per\_questionnaire.xlsx.   
You can change the name and the format of the file changing the name in the `cat_per_questionnaire.T.to_excel()` function.
  
⚠️ If you need it, save the excel file on your local computer : these online file will be deleted as soon as you quit this page!

## Distribution across the categories of the symptoms measured by each questionnaire¶

(i.e. same thing as before, but normalized by questionnaire (sum across lines equals 1)).

In [47]:

```
if df.shape[0] != df['Category'].isnull().sum() : 
  fig = px.imshow(pd.DataFrame(np.round(normalize(cat_per_questionnaire,norm='l1').T*100,1), # for printing text, we round the percentages to 1 decimal
                              columns = cat_per_questionnaire.index,
                              index= cat_per_questionnaire.columns),
                  text_auto=True, # add the text
                  color_continuous_scale= 'Portland'# more color palettes available here : https://plotly.com/python/builtin-colorscales/
  )
  fig.update_xaxes(side="top") # xaxis on top of the figure
  fig.update_layout(
    autosize=False,
    width=800,
    height=400
    )
  fig.write_image("figure3_heatmap.pdf") # writing the figure in a file
  fig.show() # showing the figure
else : 
  print('No category in this dataframe !')
```

Figure 3 has been save in the online folder (📁 symbol on the left) under the name figure3\_heatmap.pdf. You can change the name and the format of the file changing the name in the `fig.write_image()` function.
  
⚠️ If you need it, save the figure on your local computer : these online file will be deleted as soon as you quit this page!

---

# 2. Analysis and data vizualisation of content analysis Figure¶

## Changing shape of data¶

In [48]:

```
# changing the shape of data
df_col = df.melt(id_vars=['Category','Ab', 'Symptom'], value_vars=col).copy()
df_col.head()
```

Out[48]:

|  | Category | Ab | Symptom | variable | value |
| --- | --- | --- | --- | --- | --- |
| 0 | SLEEPINESS SYMPTOMS | S01 | Daytime sleepiness | ICSD | 1 |
| 1 | INSOMNIA SYMPTOMS | S06 | Insomnia initiating | ICSD | 1 |
| 2 | INSOMNIA SYMPTOMS | S07 | Insomnia maintaining | ICSD | 1 |
| 3 | RESPIRATORY SYMPTOMS | S10 | Breath abnormalities complaint | ICSD | 1 |
| 4 | RESPIRATORY SYMPTOMS | S12 | Snoring | ICSD | 1 |

## Figure¶

If you want to analyse custom data, you will have to set the variable `max_radius` so that the figure have the desired look !

In [49]:

```
###
# PARAMETERS
###
if experiment == "Fried2017" :
    max_radius = 13
elif experiment == "GauldMartin2022" : 
    max_radius = 20 # maximum radius on the final figure
else : 
    max_radius = 100 # <- /!\ value to set /!\ !!!
min_radius = 6.2 # minimum intern radius

# dictionnary questionnaire <->  radius of the circles
dic = {}
i = max_radius
for scale in df_col.variable.unique() : 
    dic[scale] = i
    i-=1

# dictionnary scale <-> color
palette = px.colors.qualitative.Pastel # choice of the palette. More choice in the following documentation : https://plotly.com/python/builtin-colorscales/
i = 0
dic_color = {}
# circular attribution of the colors (at the end of the palette, we go back to the beginning)
for scale in df_col['variable'].unique() : 
    if i>len(palette)-1 : 
        i = 0
    dic_color[scale] =palette[i]
    i+=1

###
# FIGURE
###
    
fig = go.Figure()
# transparency plot with all the symptoms to set their order in the plot
fig.add_trace(go.Scatterpolar(
            r = [0 for k in range(len(df.index))], # list of radiuses
            theta = df.Ab, # list of angles
            mode = 'markers',
            showlegend = False, # no legend thanks
        opacity = 0.0, # everything transparent !
    ))

if df.shape[0] == df['Category'].isnull().sum() : 
    df_col.loc[:,'Category'] = ""

### specific symptoms (value == 1)
df_spe = df_col[df_col.value == 1].copy() # we isolate only the specific symptoms
for scale in df_spe.variable.unique() : 
    temp = df_spe[df_spe.variable==scale] # dataframe with the data of each scale
    fig.add_trace(go.Scatterpolar(
            r = [dic[scale] for k in range(temp.shape[0])], # constant radius corresponding to the dictionnary value
            theta = temp.Ab, # angle = symptom 
            mode = 'markers',
        name = scale, # name in the legend
        hoverinfo="text", # type of hover. 'text' means that we design it by hand.
        hovertext= "Scale: "+scale+"<br>"+"Sympt.:"+temp.Symptom + "<br>Specific"+"<br>Category: "+temp.Category, # \n is <br> (html)
        # if category column in not empty
        
        opacity = 1.0,
        marker=dict( #property of the markers
            color = dic_color[scale], # color depending on the scale 
            symbol = "circle", # we want filled circles
            line=dict( # property of the line of the markers
                width=0 # we do not want line !
            ))
    ))

### component symptoms (=2)
df_comp = df_col[df_col.value == 2].copy()# we isolate only the compound symptoms
for scale in df_comp.variable.unique() : 
    temp = df_comp[df_comp.variable==scale] # dataframe with the data of each scale
    fig.add_trace(go.Scatterpolar(
            r = [dic[scale] for k in range(temp.shape[0])], # constant radius corresponding to the dictionnary value
            theta = temp.Ab,  # angle = symptom 
            mode = 'markers',
        hoverinfo="text",
        hovertext= "Scale: "+scale+"<br>"+"Sympt.:"+temp.Symptom + "<br>Compound"+"<br>Category: "+temp.Category, # \n is <br> (html)
        showlegend = False, # no legend
        marker=dict( # properties of the markers
            color = 'white', # white circle with color line
            symbol = "circle",
            line=dict(
                color=dic_color[scale], # color of the line
                width=1, # width of the line
         ))
    ))
    i+=1
    
### white circle in the center
fig.add_trace(go.Scatterpolar(
    r=[min_radius for k in range (len(df.Ab))], # radius of the circle = min_radius (set before)
    theta=df.Ab, # all angles
    fill='toself',
    fillcolor = "white", # color of the circle
    showlegend = False, # no legend
    line=dict(
    color="white",
    width=0, # no line
        ))
)


### Set options common to all traces with fig.update_traces

fig.update_polars(bgcolor='white')
fig.update_layout(
    autosize=True, # to allow or not autosize
    width=600, # width of the figure
    height=500, # height of the figure
    paper_bgcolor = 'rgba(0,0,0,0)', plot_bgcolor= 'rgba(0,0,0,0)',# background color
    polar = dict( #options for the polar plot
          radialaxis = dict(visible = True, # allowing radius lines  
                            color="lightgrey", # color of the lines
                            gridcolor = "lightgrey", # color of the grid
                            linecolor="lightgrey", #color of the lines
                            gridwidth = 1, # step in the grid
                            range=[0, max_radius+1], # range of the grid
                            dtick=1, # step in the grid
                            showgrid =True, # showing the grid
                            layer="below traces", # put the grid below traces
                            tickfont_color ='rgba(0,0,0,0)'),# putting tickfont into white to make them disappear
          angularaxis = dict(
        gridcolor = "lightgrey", # color of the angular grid
        tickfont_size=7, # font size of labels (ex. "S01")
        rotation=90, # start position of angular axis 
        direction="counterclockwise" # changin direction to align with Fried et al. 
        )),
        legend = dict(font = dict(size = 10, color = "black")) # size and color of the legend
)


fig.write_image("figure4_radial.pdf") #saving figure into a file
fig.show() # showing figure
```

The figure has been save in the online folder (📁 symbol on the left) under the name figure4\_radial.pdf. You can change the name and the format of the file changing the name in the `fig.write_image()` function.
  
⚠️ If you need it, save the figure on your local computer : these online file will be deleted as soon as you quit this page!

## Overlap between questionnaires - Jaccard Index¶

In order to estimate the overlap between the symptoms measured by the questionnes, calculate the Jaccard index, which is defined as the number of symmtoms that are measured by both questionnaires, divided by the number of unique symptoms measured both questionnaires.

### Jaccard index of symptom for each pair of questionnaire¶

First, we compute the Jaccard index for each pair of questionnaires and plot it using a heatmap.

In [50]:

```
###
# Computing the table
###
jaccard_table = pd.DataFrame(np.zeros((df.shape[1]-4,df.shape[1]-4)), index = df.columns[3:-1], columns = df.columns[3:-1]) # df.columns[3:-1] : questionnaires without header
for questionnaire1 in df.columns[3:-1] : 
  for questionnaire2 in df.columns[3:-1] : 
    jaccard_table.loc[questionnaire1, questionnaire2] = jaccard_score(df[questionnaire1]>=1, df[questionnaire2]>=1)
    
display(jaccard_table)
jaccard_table.to_excel("table3_jaccard_pairs.xlsx")
```

|  | ICSD | DSM | SDQ | Sleep50 | ASQ | SDS-CL-25 | HSDQ | PSQI | ISDI | GSAQ | SDS-CL-17 | SSC | BNSQ | OSQ |
| --- | --- | --- | --- | --- | --- | --- | --- | --- | --- | --- | --- | --- | --- | --- |
| ICSD | 1.000000 | 0.723404 | 0.553571 | 0.500000 | 0.436364 | 0.500000 | 0.423077 | 0.333333 | 0.365385 | 0.352941 | 0.416667 | 0.269231 | 0.254902 | 0.230769 |
| DSM | 0.723404 | 1.000000 | 0.480000 | 0.511111 | 0.434783 | 0.550000 | 0.525000 | 0.404762 | 0.414634 | 0.365854 | 0.571429 | 0.292683 | 0.342105 | 0.307692 |
| SDQ | 0.553571 | 0.480000 | 1.000000 | 0.644444 | 0.636364 | 0.478261 | 0.522727 | 0.382979 | 0.488372 | 0.377778 | 0.355556 | 0.372093 | 0.325581 | 0.357143 |
| Sleep50 | 0.500000 | 0.511111 | 0.644444 | 1.000000 | 0.571429 | 0.631579 | 0.605263 | 0.404762 | 0.487179 | 0.400000 | 0.486486 | 0.394737 | 0.378378 | 0.378378 |
| ASQ | 0.436364 | 0.434783 | 0.636364 | 0.571429 | 1.000000 | 0.463415 | 0.512821 | 0.425000 | 0.473684 | 0.421053 | 0.358974 | 0.416667 | 0.361111 | 0.441176 |
| SDS-CL-25 | 0.500000 | 0.550000 | 0.478261 | 0.631579 | 0.463415 | 1.000000 | 0.527778 | 0.394737 | 0.368421 | 0.388889 | 0.750000 | 0.382353 | 0.406250 | 0.363636 |
| HSDQ | 0.423077 | 0.525000 | 0.522727 | 0.605263 | 0.512821 | 0.527778 | 1.000000 | 0.368421 | 0.645161 | 0.484848 | 0.454545 | 0.437500 | 0.375000 | 0.466667 |
| PSQI | 0.333333 | 0.404762 | 0.382979 | 0.404762 | 0.425000 | 0.394737 | 0.368421 | 1.000000 | 0.400000 | 0.424242 | 0.393939 | 0.419355 | 0.500000 | 0.448276 |
| ISDI | 0.365385 | 0.414634 | 0.488372 | 0.487179 | 0.473684 | 0.368421 | 0.645161 | 0.400000 | 1.000000 | 0.483871 | 0.363636 | 0.535714 | 0.322581 | 0.518519 |
| GSAQ | 0.352941 | 0.365854 | 0.377778 | 0.400000 | 0.421053 | 0.388889 | 0.484848 | 0.424242 | 0.483871 | 1.000000 | 0.387097 | 0.464286 | 0.300000 | 0.392857 |
| SDS-CL-17 | 0.416667 | 0.571429 | 0.355556 | 0.486486 | 0.358974 | 0.750000 | 0.454545 | 0.393939 | 0.363636 | 0.387097 | 1.000000 | 0.379310 | 0.407407 | 0.357143 |
| SSC | 0.269231 | 0.292683 | 0.372093 | 0.394737 | 0.416667 | 0.382353 | 0.437500 | 0.419355 | 0.535714 | 0.464286 | 0.379310 | 1.000000 | 0.333333 | 0.440000 |
| BNSQ | 0.254902 | 0.342105 | 0.325581 | 0.378378 | 0.361111 | 0.406250 | 0.375000 | 0.500000 | 0.322581 | 0.300000 | 0.407407 | 0.333333 | 1.000000 | 0.416667 |
| OSQ | 0.230769 | 0.307692 | 0.357143 | 0.378378 | 0.441176 | 0.363636 | 0.466667 | 0.448276 | 0.518519 | 0.392857 | 0.357143 | 0.440000 | 0.416667 | 1.000000 |

Table 3 has been save in the online folder (📁 symbol on the left) under the name table3\_jaccard\_pairs.xlsx.   
You can change the name and the format of the file changing the name in the `jaccard_table.to_excel()` function.
  
⚠️ If you need it, save the excel file on your local computer : these online file will be deleted as soon as you quit this page!

In [51]:

```
###
# Plotting it as a heatmap
###
fig = px.imshow(pd.DataFrame(np.round(jaccard_table,3), # rounding values for the plot
                             index = jaccard_table.index,
                             columns= jaccard_table.columns),
                text_auto=True, # annotating values in the plot
                color_continuous_scale= 'Portland'# more color palettes available here : https://plotly.com/python/builtin-colorscales/
)
fig.update_xaxes(side="top")
fig.update_layout(
    autosize=False,
    width=800,
    height=800
    )
fig.write_image("figure5_heatmap_jaccard.pdf") # writting the figure into a file
fig.show() # showing figure
```

The figure has been save in the online folder (📁 symbol on the left) under the name figure5\_heatmap\_jaccard.pdf.   
You can change the name and the format of the file changing the name in the `fig.write_image()` function.
  
⚠️ If you want it, save the figure on your local computer : these online file will be deleted as soon as you quit this page!

### Avg. Jaccard index¶

Then, we compute the average of Jaccard index for each questionnaire with other questionnaires (excluding the references).

In [52]:

```
jaccard = pd.DataFrame(np.zeros((len(df.drop(header+['sum_symptoms'],axis = 1).columns),1)), index = df.drop(header+['sum_symptoms'],axis = 1).columns, columns=['Avg. Jaccard Index'])
for questionnaire in df.drop(header+['sum_symptoms'], axis=1).columns : 
  jaccard.loc[questionnaire, 'Avg. Jaccard Index'] = jaccard_table.drop(references+[questionnaire], axis = 1).loc[questionnaire, :].mean()
display(jaccard)
jaccard.to_excel("table4_jaccard_average_questionnaires.xlsx")
print("Average Jaccard index (wo references): "+str(np.round(float(jaccard.mean()),5)) +" (sd: "+str(np.round(float(jaccard.std()),4))+ ")" )
```

|  | Avg. Jaccard Index |
| --- | --- |
| SDQ | 0.449209 |
| Sleep50 | 0.489331 |
| ASQ | 0.461972 |
| SDS-CL-25 | 0.468665 |
| HSDQ | 0.490976 |
| PSQI | 0.414701 |
| ISDI | 0.462467 |
| GSAQ | 0.411356 |
| SDS-CL-17 | 0.426736 |
| SSC | 0.415941 |
| BNSQ | 0.375119 |
| OSQ | 0.416406 |

```
Average Jaccard index (wo references): 0.44024 (sd: 0.0356)
```

Table 4 has been save in the online folder (📁 symbol on the left) under the name table4\_jaccard\_average\_questionnaires.xlsx.   
You can change the name and the format of the file changing the name in the `jaccard.to_excel()` function.
  
⚠️ If you need it, save the excel file on your local computer : these online file will be deleted as soon as you quit this page!

### Correlation between the number of symptoms and the average Jacquart index for each questionnaire¶

In [53]:

```
correlations = jaccard.join(sympt_per_questionnaire)
display(correlations)
print("Correlation between Jaccard Index and number of specific symptoms: ",spearmanr(correlations['Avg. Jaccard Index'], correlations['Specific symptoms']))
print("Correlation between Jaccard Index and number of compound symptoms: ",spearmanr(correlations['Avg. Jaccard Index'], correlations['Compound symptoms']))
print("Correlation between Jaccard Index and total number of symptoms: ",spearmanr(correlations['Avg. Jaccard Index'], correlations['Total']))
```

|  | Avg. Jaccard Index | Specific symptoms | Compound symptoms | Total |
| --- | --- | --- | --- | --- |
| SDQ | 0.449209 | 32 | 8 | 40 |
| Sleep50 | 0.489331 | 31 | 3 | 34 |
| ASQ | 0.461972 | 23 | 9 | 32 |
| SDS-CL-25 | 0.468665 | 20 | 8 | 28 |
| HSDQ | 0.490976 | 15 | 12 | 27 |
| PSQI | 0.414701 | 13 | 12 | 25 |
| ISDI | 0.462467 | 16 | 8 | 24 |
| GSAQ | 0.411356 | 4 | 18 | 22 |
| SDS-CL-17 | 0.426736 | 13 | 8 | 21 |
| SSC | 0.415941 | 17 | 2 | 19 |
| BNSQ | 0.375119 | 11 | 6 | 17 |
| OSQ | 0.416406 | 12 | 5 | 17 |

```
Correlation between Jaccard Index and number of specific symptoms:  SpearmanrResult(correlation=0.6619975125797373, pvalue=0.019019455833880407)
Correlation between Jaccard Index and number of compound symptoms:  SpearmanrResult(correlation=-0.0035657479116033464, pvalue=0.9912250660088198)
Correlation between Jaccard Index and total number of symptoms:  SpearmanrResult(correlation=0.6584948802380455, pvalue=0.019892133524913664)
```

### Jaccard index of symtpoms for each pair of questionnaire for each category¶

Computing the same metric (average of average) for each category of questionnaires.

In [54]:

```
if df.shape[0] != df['Category'].isnull().sum() : 
  res = pd.DataFrame(np.zeros((len(df.Category.unique()),1)), index = df.sort_values(by="Ab").Category.unique(), columns=['Avg. Jaccard Index'])
  for category in df.Category.unique() : 
      df_category = df.drop(header+['sum_symptoms'],axis = 1)[df.Category==category]
      df_category = df_category.iloc[:,(df_category.sum(axis = 0)!=0.0).to_numpy()] # we keep only the questionnaire with at least 1 symptom
      liste_avg = []
      for questionnaire1 in df_category.columns : 
        liste = []
        for questionnaire2 in df_category.columns : 
          if questionnaire1!= questionnaire2 :
            liste.append(jaccard_score(df_category[questionnaire1]>=1, df_category[questionnaire2]>=1))
        liste_avg.append(np.mean(liste))
      res.loc[category, 'Avg. Jaccard Index'] = np.mean(liste_avg)
  display(res)
  res.to_excel("table5_jaccard_categories.xlsx")

else : 
  print("Category is empty")
```

|  | Avg. Jaccard Index |
| --- | --- |
| SLEEPINESS SYMPTOMS | 0.606061 |
| INSOMNIA SYMPTOMS | 0.791667 |
| RESPIRATORY SYMPTOMS | 0.757576 |
| PSYCHIATRIC SYMPTOMS | 0.444444 |
| BEHAVIORAL SYMPTOMS DURING SLEEP | 0.438413 |
| MOTOR SYMPTOMS | 0.460216 |
| GENERAL SYMPTOMS | 0.398148 |
| SLEEP PERIOD SYMPTOMS | 0.353030 |
| NON OTHERWISE SPECIFIED | 0.372424 |

Table 5 has been save in the online folder (📁 symbol on the left) under the name table5\_jaccard\_categories.xlsx.   
You can change the name and the format of the file changing the name in the `res.to_excel()` function.
  
⚠️ If you need it, save the excel file on your local computer : these online file will be deleted as soon as you quit this page!

---

# Export to html¶

You have reached the end of this notebook.
If you want to save the whole page, you can download it to html with dynamic figures:

> - "File" → "Save and Export Notebook as" → "HTML"
